# Independent and combined association of milk tea and takeaway food consumption on depression, anxiety, and comorbid symptoms among Chinese university students

**DOI:** 10.3389/fnut.2025.1693936

**Published:** 2026-01-14

**Authors:** Juncheng Zhu, Jinkui Lu, Lijun Tang, Helin Jin, Cong Liu, Xinping Yuan, Hao Luo, Zhixiu He, Jiancai Song, Shuaicheng Luo

**Affiliations:** 1 School of Physical Education, Hainan Normal University, Haikou, Hainan, China; 2 School of Physical Education, Shangrao Normal University, Shangrao, Jiangxi, China; 3 School of Physical Education, Shanghai Normal University, Shanghai, China; 4 School of Physical Education and Health, Shanghai University of International Business and Economics, Shanghai, China; 5 Physical Education College, Jiangxi Normal University, Nanchang, Jiangxi, China; 6 School of Teacher Education, Shangrao Normal University, Shangrao, Jiangxi, China; 7 Physical Education Teaching and Research Section, School of Marxism, Jiangxi Medical College, Shangrao, Jiangxi, China

**Keywords:** milk tea consumption, takeaway food consumption, combined association, depression, anxiety, Chinese university students

## Abstract

**Background:**

Milk tea and takeaway food consumption are increasingly prevalent among Chinese university students and may contribute to adverse mental health outcomes. However, the combined association of these dietary behaviors on psychological symptoms remain unclear.

**Methods:**

A multicenter cross-sectional survey was conducted from September to November 2023 among 15,440 students from seven universities in four provinces of China. Single-factor associations between psychological symptom types and milk tea/takeaway food consumption were examined using the Chi-square test. Multivariate logistic regression was then employed to assess independent associations, adjusting for demographic and lifestyle confounders. Finally, interaction models were used to explore the combined association of milk tea and takeaway food consumption, as well as the impact of specific consumption combinations, on depression symptoms, anxiety symptoms, and their comorbidity. Odds ratios (OR) and 95% confidence intervals (CI) were calculated.

**Results:**

Milk tea consumption was associated with an increased likelihood of only anxiety symptoms (OR = 1.33, 95% CI: 1.18–1.50) and comorbidity (OR = 1.16, 95% CI: 1.06–1.26). Takeaway food consumption was associated with only anxiety symptoms (OR = 1.23, 95% CI: 1.09–1.40). Students who consumed both milk tea and takeaway food had a higher risk of only anxiety symptoms (OR = 1.67, 95% CI: 1.43–1.95) and comorbidity (OR = 1.24, 95% CI: 1.11–1.38). Several specific consumption combinations—particularly cream cap milk tea with grilled/deep-fried skewers—were strongly linked to comorbidity (OR = 4.25, 95% CI: 3.61–5.02).

**Conclusion:**

Both milk tea and takeaway food consumption are independently associated with higher risks of anxiety symptoms and comorbid depression–anxiety among Chinese university students, with a significant combined association observed. High-fat, high-sugar milk tea combined with deep-fried or stir-fried takeaway food poses the greatest risk, highlighting the need for targeted dietary interventions to mitigate psychological symptom burden in this population.

## Introduction

Depression and anxiety are among the most prevalent mental health disorders worldwide, particularly in adolescents and young adults (1). According to the Global Burden of Disease 2021 study, the prevalence of depressive and anxiety disorders increases substantially from early adolescence (10–19 years: 2.43% and 4.94%, respectively) to young adulthood (20–24 years: 4.79% and 5.80%) (2). These trends may be largely attributed to academic pressure, social relationship challenges, and the stress of job seeking. A systematic review from the United Kingdom reported that high academic pressure was strongly associated with poor mental health. Similarly, a cross-sectional study of 10,676 Chinese university students found that social support played an important role in mitigating psychological distress (3). Notably, depression and anxiety are highly comorbid, with many students experiencing both simultaneously (4). For instance, one study during the COVID-19 pandemic reported that the prevalence of depressive symptoms, anxiety symptoms, and their comorbidity among Chinese university students was 37.0%, 24.9%, and 20.9%, respectively (5). Prolonged exposure to these conditions can severely impair health and, in severe cases, lead to suicidal behavior, underscoring the urgent need to address university students’ mental health.

The contributing factors of depression and anxiety are diverse, including environmental (6), genetic (7), lifestyle (8), and socio-demographic factors (9). Among lifestyle factors, dietary habits have received growing research attention as potential modifiable risk factors for psychological symptoms in adolescents (10). A multicenter cross-sectional study of 14,340 Chinese university students found that low physical activity and frequent takeaway food consumption were positively associated with the comorbidity of depression and anxiety, with a significant combined association (11). Another study of 5,281 Beijing college students reported that milk tea consumption was common, and higher intake was associated with increased risks of depressive symptoms, anxiety symptoms, and suicidal ideation (12). Evidence also suggests that healthy dietary patterns—such as higher fruit and vegetable intake—are linked to better mental health, whereas unhealthy diets, characterized by frequent fast food or sugar-sweetened beverage consumption, increase the risk of depression, anxiety, and stress (13). For example, a Korean study found that individuals with high intake of both fast food and sugar-sweetened beverages had 1. 28-, 1. 59-, and 1.32-fold higher odds of stress, depression, and suicidal ideation, respectively (14). Similarly, a study in Hong Kong young adults (aged 18–27 years) showed that diets high in fat, sugar, and sodium were associated with greater odds of depressive and anxiety symptoms (15). Notably, Chinese university students frequently engage in unhealthy eating behaviors and have low nutrition literacy (16), such as skipping breakfast (17), eating late at night (18), eating irregularly (19), and preferring fast food, barbecue, or milk tea (20, 21). The popularity and convenience of takeaway food on campus further exacerbate the consumption of unhealthy options (22). Therefore, universities should prioritize interventions to improve students’ dietary behaviors and mental health.

Currently, previous studies have explored the associations between specific dietary behaviors [i.e., breakfast (23), sugar-sweetened beverage (24), fast food (25), vegetable (26), soybean product (27)] and psychological issues among university students (28). However, most studies have examined the relationship between individual dietary behaviors and mental health in isolation, and the combined association of multiple dietary factors remain unclear (29). Moreover, several studies have found that typical unhealthy dietary behaviors among university students (i.e., sugar-sweetened beverage, fast food, and takeaway food consumption) are adversely associated with mental health (30). Nevertheless, the subcategories of food or beverage types have not been considered in these studies (31). At present, milk tea and takeaway food are popular among students on campus due to their pleasant taste, variety of choices, and convenience (32). However, limited research has examined the association between milk tea and takeaway food consumption and psychological issues (33), and the potential combined association between them remain unclear. To bridge these research gaps, we conducted a multicenter cross-sectional study to investigate the associations of milk tea and takeaway food consumption with psychological symptoms among Chinese university students. The hypotheses of the present study are as follows:

Hypothesis 1 (H1): Milk tea and takeaway food consumption are positively associated with depression symptoms, anxiety symptoms, and their comorbidity among university students.

Hypothesis 2 (H2): Milk tea and takeaway food consumption exert a positive combined association on depression symptoms, anxiety symptoms, and their comorbidity among university students.

Hypothesis 3 (H3): The combined association of milk tea and takeaway food consumption on depression symptoms, anxiety symptoms, and their comorbidity vary across different combinations of consumption types.

## Methods and participants

### Sample estimation

The minimum sample size for this epidemiological cross-sectional study was calculated using the formula: “*n* = μ_α_^2^ρ (1−ρ)/δ^2^.” In this formula, ρ represents the prevalence rate, δ denotes the admissible error (δ = 0.1 × ρ), μ_α_ is the critical value from the standard normal distribution corresponding to a 95% confidence level (μ_α_ = 1.96). Based on the comorbidity rate of depression and anxiety (23.75%) reported in the study by Zhang et al. (11), the calculated minimum sample size was 1,234. Considering an anticipated 10% sample loss and 10% invalid responses, the final minimum required sample size was 1,481.

### Sampling method

Following the principle of convenience sampling, we surveyed seven partner universities located in Shanghai, Jiangxi, Hubei, and Shanxi provinces, China, between September and November 2023. First, in each participating university, six departments (three liberal arts departments and three science departments) were randomly selected. Second, four grades were chosen within each department. Third, three administrative classes were randomly selected from each grade. However, as most students in the junior and senior grades were engaged in internships, additional freshman students were recruited to ensure the sample size could achieve the desired statistical power. The average population size of each administrative class was approximately 35–40 students. In total, the present study involved seven universities, 42 departments, 504 administrative classes, and approximately 17,000 students. Before the survey, students were informed of the study’s purpose and assured that participation was voluntary. Written informed consent was obtained from all participants. The study was approved by the Ethics Committee of Jiangxi Medical School (approval number: R2022-2).

### Participants

**FIGURE 1:**
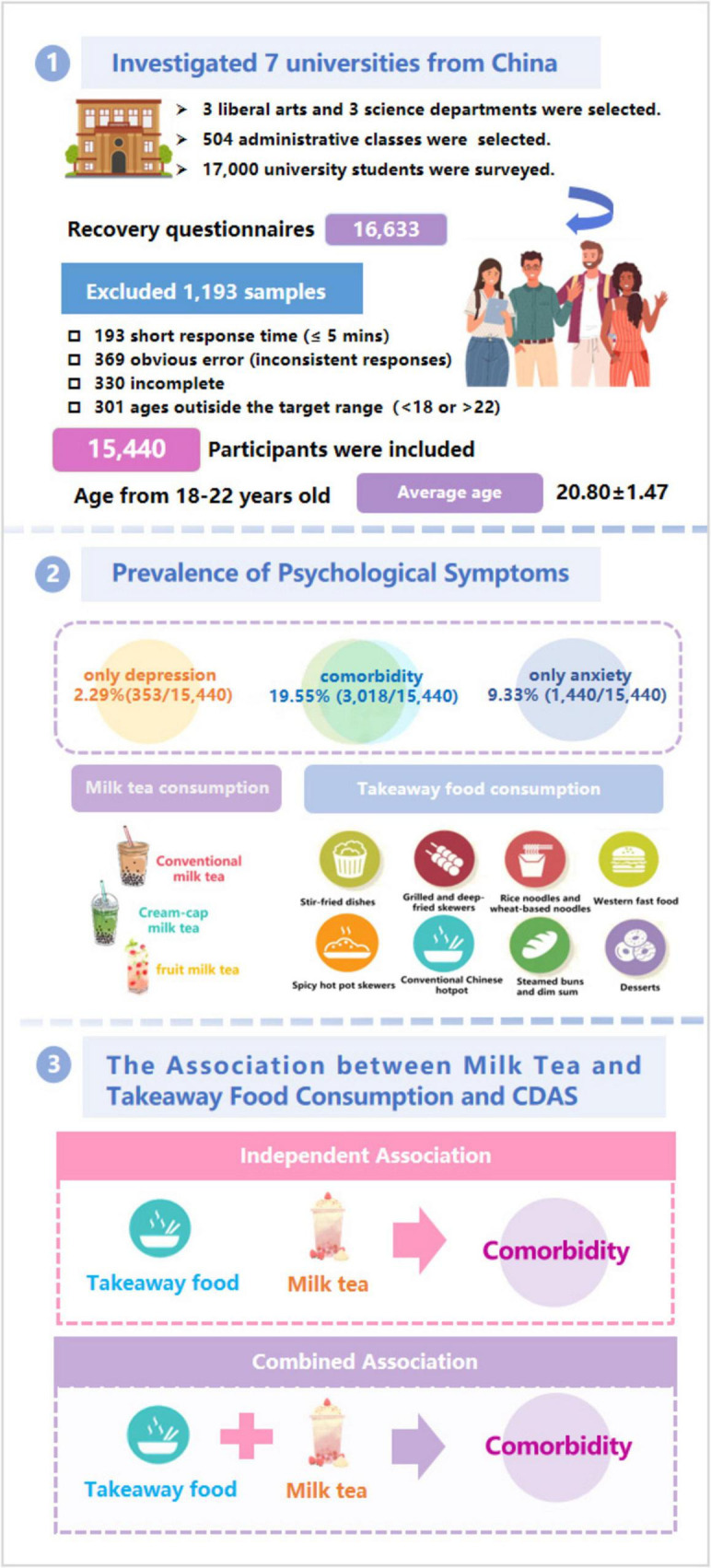
The flow chart.

In the present study, participants were Chinese university students aged 18–22 years. The process of online questionnaire distribution and participant inclusion was as follows. The questionnaires were administered via the Wenjuanxing platform. A total of 17,000 questionnaires were distributed, and 16,633 were returned, yielding a recovery rate of 97.84%. Among the collected questionnaires, 193 were completed in less than five minutes, 369 contained obvious errors (e.g., inconsistent responses), 330 were incomplete, and 301 reported ages outside the target range. These questionnaires were excluded, resulting in 15,440 valid questionnaires, with a validity rate of 92.83% (15,440/16,633) (see Figure 1). Ultimately, 15,440 university students (aged 18–22 years, mean age 20.80 ± 1.47 years) were included in the analysis. Among them, 6,171 (39.97%) were male and 9,269 (60.03%) were female; 6,457 (41.82%) were from urban areas, and 8,983 (58.18%) were from rural areas (see Table 1).

**TABLE 1:** The basic information of participants.

Groups	Overall (*N* = 15,440)	Male (*N* = 6,171)	Female (*N* = 9,269)
Age	20.80 ± 1.42	20.80 ± 1.47	20.80 ± 1.38
**Household registration**			
Urban	6,457	2,475 (40.1)	3,982 (43.0)
Rural	8,983	3,696 (59.9)	5,287 (57.0)
**Grade**			
Freshman	9,040	3,920 (63.5)	5,120 (55.2)
Sophomore	3,022	1,181 (19.1)	1,841 (19.9)
Junior	2,066	629 (10.2)	1,437 (15.5)
Senior	1,312	441 (7.2)	871 (9.4)
**Father’s education**			
Primary school or below	2,288	951 (15.4)	1,337 (14.4)
Middle school	6,078	2,514 (40.7)	3,564 (38.5)
High school/vocational school	4,409	1,709 (27.7)	2,700 (29.1)
College or above	2,665	997 (16.2)	1,668 (18.0)
**Mother’s education**			
Primary school or below	3,978	1,664 (27.0)	2,314 (25.0)
Middle school	5,597	2,280 (36.9)	3,317 (35.8)
High school/vocational school	3,752	1,441 (23.4)	2,311 (24.9)
College or above	2,113	786 (12.7)	1,327 (14.3)
**Annual family income**			
≤50,000	7,177	2,859 (46.3)	4,318 (46.6)
50,001–100,000	3,096	1,281 (20.8)	1,815 (19.6)
100,001–300,000	4,065	1,573 (25.5)	2,492 (26.9)
≥300,001	1,102	458 (7.4)	644 (6.9)
**BMI status**			
Underweight	1,061	364 (5.9)	697 (7.5)
Normal	10,886	3,953 (64.1)	6,933 (74.8)
Overweight	1,982	1,213 (19.6)	769(8.3)
Obese	1,511	641 (10.4)	870 (9.4)
**MVPA duration/week**			
<75 min	8,840	2,859 (46.3)	5,981 (64.5)
75∼150 min	119	35 (0.6)	84 (0.9)
151∼225 min	484	145 (2.3)	339 (3.7)
>225 min	5,597	3,132 (50.8)	2,865 (30.9)
**Sedentary behaviors duration/day**			
<4.0 h	9,682	4,128 (66.9)	5,554 (59.9)
4.0∼6.0 h	4,246	1,586 (25.7)	2,660 (28.7)
>6 h	1,512	457(7.4)	1,055 (11.4)
**Sleep duration/day**			
<7.0 h	2,483	1,054 (17.1)	1,429 (15.4)
7.0∼8.0 h	7,236	2,898 (46.9)	4,338 (46.8)
8.1∼9.0 h	4,196	1,646 (26.7)	2,550 (27.5)
>9.0 h	1,525	573(9.3)	952(10.3)

The values inside parentheses () are proportions.

### Socio-demographic information

Socio-demographic characteristics were collected via self-reported questionnaires, including age, sex (male, female), household registration (urban, rural), grade (freshman, sophomore, junior, senior), father’s education (primary school or below, middle school, high school/vocational school, college or above), mother’s education (primary school or below, middle school, high school/vocational school, college or above), annual family income (≤50,000, 50,000–100,000, 100,001–300,000, ≥300,001), and BMI status (underweight, normal, overweight, obese). BMI was calculated as weight (kg) divided by height squared (m^2^), measured during physical education classes before the survey. BMI categories were determined according to the WHO classification criteria (2006).

### Depression and anxiety symptom assessment

Depression and anxiety symptoms were assessed using the Chinese version of the Depression Anxiety and Stress Scale (DASS-21). This scale contains 21 items across three dimensions assessing depression, anxiety, and stress symptoms. Each item has four response options: 0 (“Did not apply to me at all”), 1 (“Applied to me to some degree or some of the time”), 2 (“Applied to me to a considerable degree or a good part of the time”), and 3 (“Applied to me very much or most of the time”). Scores for each dimension were summed and then multiplied by two, as per DASS-21 scoring guidelines. In this study, a depression score ≥ 10 indicated depressive symptoms, and an anxiety score ≥ 8 indicated anxiety symptoms. Based on symptom type, participants were further classified into: ? only depression symptoms (depression score ≥ 10 and anxiety score < 8), ? only anxiety symptoms (depression score < 10 and anxiety score ≥ 8), and ? comorbidity (depression score ≥ 10 and anxiety score ≥ 8). Besides, the Cronbach’s α coefficients for the depression and anxiety dimensions were 0.905 and 0.932, respectively.

### Milk tea and takeaway food behavior assessment

Dietary behaviors were assessed via self-reported questionnaires on milk tea and takeaway food consumption in the past seven days.

#### Milk tea behavior assessment

Milk tea consumption was evaluated using a semi-quantitative food frequency questionnaire adapted from previous studies. The first question, “Have you consumed milk tea in the past seven days?”, identified whether participants had engaged in this behavior. This 7-day window was pre-specified to minimize recall bias and to align with common practice in national dietary surveillance and recent university-based food frequency questionnaires. According to prior literature and the findings of the present survey (20), milk tea was categorized into conventional milk tea, fruit milk tea, and cream cap milk tea. The second question assessed the frequency of consumption for each type, with four options: “No consumption,” “1–4 bottles,” “5–9 bottles,” and “≥10 bottles.” In addition, individuals reporting milk tea consumption of more than one bottle were classified as having the corresponding type of milk tea consumption behavior.

#### Takeaway food behavior assessment

Similarly, takeaway food consumption was assessed with two items. The first question, “Have you consumed takeaway food in the past seven days?”, identified relevant behaviors. The second question investigated the frequency of consumption of eight popular takeaway food types in China: stir-fried dishes, grilled and deep-fried skewers, rice noodles and wheat-based noodles, Western fast food, spicy hot pot skewers (Malatang), conventional Chinese hotpot (Huoguo), steamed buns and dim sum, and desserts. Each item offered nine options, ranging from “0 times” to “more than 8 times per week.” Based on previous literature and participants consumption situations, the groups as follow “≤1 time,” “2–4 times,” “5–7 times,” and “≥8 times” (34). In addition, participants who reported consuming takeaway food more than once were categorized as having the corresponding type of takeaway food consumption behavior.

### Quality control

The survey was conducted by trained university teachers and students. Before data collection, all investigators received standardized training. The online questionnaire was distributed during class meetings via a controlled link to ensure each student could complete it only once. Investigators were available to answer questions during the survey. Completed questionnaires were collected and verified on-site. The survey took approximately 40 minutes to complete, and each participant received a small doll pendant as a token of appreciation.

### Statistical analysis

Continuous variables were presented as means ± standard deviations (M ± SD), and categorical variables were expressed as numbers and percentages. The Chi-square test was used to compare differences in types of psychological symptoms, milk tea consumption behaviors, and takeaway food consumption behaviors by sex. In addition, Chi-square tests were also applied to examine the univariate associations between types of psychological symptoms and both milk tea and takeaway food consumption behaviors. Multivariate logistic regression models were then performed to assess the independent and combined associations of milk tea and takeaway food consumption behaviors with different types of psychological symptoms. Odds ratios (OR) and 95% confidence intervals (95% CI) were derived from the logistic regression models and visualized using forest plots. A *p*-value < 0.05 was considered statistically significant. All statistical analyses were conducted using IBM SPSS Statistics version 27.0 (IBM Corp., Armonk, NY, United States), and plots were generated using R software version 4.5.0 (R Foundation for Statistical Computing, Vienna, Austria).

## Results

### Basic characteristics of participants

A total of 15,440 students aged 18–22 years from seven universities were included in the present study. Among them, 39.97% (6,171/15,440) were male and 60.03% (9,269/15,440) were female. Rural students accounted for a larger proportion than urban students (58.18% vs. 41.82%). The distribution of participants by academic grade was as follows: freshmen, 58.55% (9,040/15,440); sophomores, 19.57% (3,022/15,440); juniors, 13.38% (2,066/15,440); and seniors, 8.50% (1,312/15,440) (Table 1).

### Prevalence of psychological symptoms and dietary behaviors

The prevalence of only depression symptoms, only anxiety symptoms, and comorbidity was 2.29% (353/15,440), 9.33% (1,440/15,440), and 19.55% (3,018/15,440), respectively. Notably, comorbidity prevalence was significantly higher in males than in females (21.6% vs. 18.2%), whereas only anxiety symptoms were more common in females than in males (10.7% vs. 7.2%) (χ^2^ = 71.52, *p* < 0.001).

Overall, 54.60% of students reported consuming milk tea in the past 7 days, with a significantly higher proportion among females than males (64.1% vs. 40.3%). Most students reported consuming 1–4 bottles per week, with fruit milk tea being the most preferred type (Table 2). Similarly, a greater proportion of females than males reported takeaway food consumption (68.9% vs. 57.8%). Females were more likely to consume stir-fried dishes, rice noodles/wheat-based noodles, spicy hot pot skewers (Malatang), and desserts, whereas males favored stir-fried dishes and rice noodles/wheat-based noodles (Table 2).

**TABLE 2:** Prevalence of psychological symptoms and dietary behaviors.

Groups	Overall (*N* = 15,440)	Male (*N* = 6,171)	Female (*N* = 9,269)	χ^2^-value	*P*-value
Depression and anxiety symptoms status				71.52	**<0.001**
Only depression	353	141 (2.3)	212 (2.3)	–	–
Only anxiety	1,440	445 (7.2)	995 (10.7)	–	–
Comorbidity	3,018	1,333 (21.6)	1,685 (18.2)	–	–
No symptoms	10,629	4,252(68.9)	6,377 (68.8)	–	–
**Milk tea consumption behaviors**					
Drinking milk tea				853.41	**<0.001**
Yes	8,430	2,484 (40.3)	5,946 (64.1)	–	–
No	7,010	3,687 (59.7)	3,323 (35.9)	–	–
**The consumption of drinking milk tea/week**					
Conventional milk tea				160.30	**<0.001**
≥10 bottle	204	118 (1.9)	86 (0.9)	–	–
5–9 bottle	194	92 (1.5)	102 (1.1)	–	–
1–4 bottle	2,769	834 (13.5)	1,935 (20.9)	–	–
No consumption	12,273	5,127 (83.1)	7,146 (77.1)	–	–
Fruit milk tea				317.68	**<0.001**
≥10 bottle	199	106 (1.7)	93 (1.0)	–	–
5–9 bottle	273	104 (1.7)	169 (1.8)	–	–
1–4 bottle	3,433	929 (15.1)	2,504 (27.0)	–	–
No consumption	11,535	5,032 (81.5)	6,503 (70.2)	–	–
Cream cap milk tea				100.12	**<0.001**
≥10 bottle	186	111 (1.8)	75 (0.8)	–	–
5–9 bottle	161	76 (1.2)	85 (0.9)	–	–
1–4 bottle	1,316	384 (6.2)	932 (10.1)	–	–
No consumption	13,777	5,600 (90.8)	8,177 (88.2)	–	–
The volume of drinking milk tea/time				933.52	**< 0.001**
Oversize cup (around 1,000 ml)	134	75 (1.2)	59 (0.6)	–	–
Large cup (around 750 ml)	2,610	827 (13.4)	1,783 (19.2)	–	–
Medium cup (around 500 ml)	4,825	1,271 (20.6)	354 (38.3)	–	–
Small cup (around 380 ml)	861	311 (5.0)	550 (5.9)	–	–
No consumption	7,010	3,687 (59.8)	3,323 (36.0)	–	–
**Takeaway food consumption behaviors**					
Eating takeaway food				199.51	**<0.001**
Yes	9,959	3,569 (57.8)	6,390 (68.9)	–	–
No	5,481	2,602 (42.2)	2,879 (31.1)	–	–
**The consumption of takeaway food/week**					
Stir-fried dishes				55.71	**<0.001**
≥8 times	386	184 (3.0)	202 (2.2)	–	–
5–7 times	945	382 (6.2)	563 (6.1)	–	–
2–4 times	3,441	1,198 (19.4)	2,243 (24.2)	–	–
≤1 times	10,668	4,407 (71.4)	6,261 (67.5)	–	–
Grilled and deep-fried skewers				11.18	**0.011**
≥8 times	109	57 (0.9)	52 (0.6)	–	–
5–7 times	280	128 (2.1)	152 (1.6)	–	–
2–4 times	1,351	530 (8.6)	821 (8.9)	–	–
≤1 times	13,700	5,456 (88.4)	8,244 (88.9)	–	–
Rice noodles and wheat-based noodles				109.23	**<0.001**
≥8 times	310	143 (2.3)	167 (1.8)	–	–
5–7 times	925	347 (5.6)	578 (6.2)	–	–
2–4 times	3,843	1,275 (20.7)	2,568 (27.7)	–	–
≤1 times	10,362	4,406 (71.4)	5,956 (64.3)	–	–
Western fast food				18.40	**<0.001**
≥8 times	110	63 (1.0)	47 (0.5)	–	–
5–7 times	386	174 (2.8)	212 (2.3)	–	–
2–4 times	2,289	904 (14.7)	1,385 (14.9)	–	–
≤1 times	12,655	5,030 (81.5)	7,625 (82.3)	–	–
Spicy hot pot skewers (Malatang)				91.75	**<0.001**
≥8 times	143	61 (1.0)	82 (0.9)	–	–
5–7 times	389	147 (2.4)	242 (2.6)	–	–
2–4 times	2,172	668 (10.8)	1,504 (16.2)	–	–
≤1 times	12,736	5,295 (85.8)	7,441 (80.3)	–	–
Conventional Chinese hotpot (Huoguo)				6.04	0.110
≥8 times	104	48 (0.8)	56 (0.6)	–	–
5–7 times	322	134 (2.2)	188 (2.0)	–	–
2–4 times	1,308	488 (7.9)	820 (8.9)	–	–
≤1 times	13,706	5,501 (89.1)	8,205 (88.5)	–	–
Steamed buns and dim sum				8.93	**0.030**
≥8 times	144	61 (1.0)	83 (0.9)	–	–
5–7 times	569	225 (3.6)	344 (3.7)	–	–
2–4 times	1,931	713 (11.6)	1,218 (13.1)	–	–
≤1 times	12,796	5,172 (83.8)	7,624 (82.3)	–	–
Desserts				238.71	**<0.001**
≥8 times	242	97 (1.6)	145 (1.5)	–	–
5–7 times	703	240 (3.9)	463 (5.0)	–	–
2–4 times	3,326	964 (15.6)	2,362 (25.5)	–	–
≤1 times	11,169	4,870 (78.9)	6,299 (68.0)	–	–

The values inside parentheses () are proportions. Bold *P* values indicate statistical significance (*P* < 0.05).

### Associations of milk tea and takeaway food consumption with psychological symptoms

**FIGURE 2:**
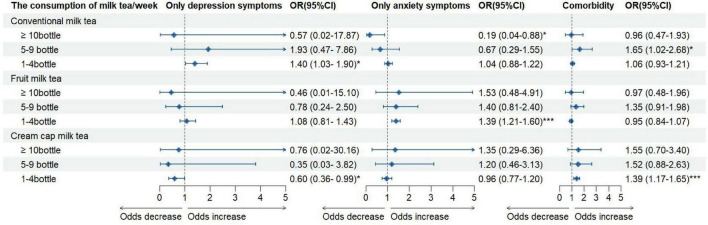
The association between milk tea consumption and psychological symptoms status. *P < 0.05; **P < 0.01; ***P < 0.001.

Univariate analyses showed that the prevalence of psychological symptom types differed significantly by milk tea and takeaway food consumption status (Table 3). In multivariate logistic regression analyses adjusting for demographic and lifestyle covariates, milk tea consumption was associated with a higher likelihood of only anxiety symptoms (OR = 1.33, 95% CI: 1.18–1.50) and comorbidity (OR = 1.16, 95% CI: 1.06–1.26; both *p* < 0.001) compared with no consumption. Takeaway food consumption was also associated with an increased likelihood of only anxiety symptoms (OR = 1.23, 95% CI: 1.09–1.40, *p* < 0.001) (Table 4). When frequency of consumption was considered, most categories of milk tea were positively associated with only depression symptoms, only anxiety symptoms, and comorbidity (OR range: 1.40–1.65; 95% CI: 1.03–2.68; all *p* < 0.05). However, some negative associations were observed: ≥10 bottles/week of conventional milk tea was inversely associated with only anxiety symptoms (OR = 0.19, 95% CI: 0.04–0.88), and 1–4 bottles/week of cream cap milk tea was inversely associated with only depression symptoms (OR = 0.60, 95% CI: 0.36–0.99) (Figure 2).

**TABLE 3:** Univariate analysis of the relationship between milk tea and takeaway food consumption and psychological symptoms.

Groups	Total	Only depression	Only anxiety	Comorbidity	χ^2^-value	*P*-value
**Milk tea consumption behaviors**						
Drinking milk tea					47.22	**<0.001**
Yes	8,430	189 (2.2)	892 (10.6)	1,711 (20.3)	–	–
No	7,010	164 (2.3)	548 (7.8)	1,307 (18.6)	–	–
**The consumption of drinking milk tea/week**						
Conventional milk tea					207.08	**<0.001**
≥10 bottle	204	1 (0.5)	5 (2.5)	76 (37.3)	–	–
5–9 bottle	194	3 (1.5)	11 (5.7)	99 (51.0)	–	–
1–4 bottle	2,769	71 (2.6)	307 (11.1)	596 (21.5)	–	–
No consumption	12,273	278 (2.3)	1,117 (9.1)	2,247 (18.3)	–	–
Fruit milk tea					203.11	**<0.001**
≥10 bottle	199	1 (0.5)	8 (4.0)	72 (36.2)	–	–
5–9 bottle	273	4 (1.5)	23 (8.4)	120 (44.0)	–	–
1–4 bottle	3,433	83 (2.4)	424 (12.4)	684 (19.9)	–	–
No consumption	11,535	265 (2.3)	985 (8.5)	2,142 (18.6)	–	–
Cream cap milk tea					242.38	**<0.001**
≥10 bottle	186	1 (0.5)	6 (3.2)	71 (38.2)	–	–
5–9 bottle	161	1 (0.6)	9 (5.6)	87 (54.0)	–	–
1–4 bottle	1,316	21 (1.6)	138 (10.5)	358 (27.2)	–	–
No consumption	13,777	330 (2.4)	1,287 (9.3)	2,502 (18.2)	–	–
**Takeaway food consumption behaviors**						
Eating takeaway food					33.26	**<0.001**
Yes	9,959	233 (2.3)	1,008 (10.1)	2,007 (20.2)	–	–
No	5,481	120 (2.2)	432 (7.9)	1,011 (18.4)	–	–
**The consumption of takeaway food/week**						
Stir-fried dishes					140.27	**<0.001**
≥8 times	386	8 (2.1)	40 (10.4)	98 (25.4)	–	–
5–7 times	945	20 (2.1)	94 (9.9)	271 (28.7)	–	–
2–4 times	3,441	76 (2.2)	354 (10.3)	800 (23.2)	–	–
≤1 times	10,668	249 (2.3)	952 (8.9)	1,849 (17.3)	–	–
Grilled and deep-fried skewers					564.55	**<0.001**
≥8 times	109	4 (3.7)	10 (9.2)	49 (45.0)	–	–
5–7 times	280	3 (1.1)	17 (6.1)	155 (55.4)	–	–
2–4 times	1,351	29 (2.1)	108 (8.0)	485 (35.9)	–	–
≤1 times	13,700	317 (2.3)	1,305 (9.5)	2,329 (17.0)	–	–
Rice noodles and wheat-based noodles					200.07	**<0.001**
≥8 times	310	10 (3.2)	26 (8.4)	95 (30.6)	–	–
5–7 times	925	23 (2.5)	76 (8.2)	290 (31.4)	–	–
2–4 times	3,843	89 (2.3)	450 (11.7)	835 (21.7)	–	–
≤1 times	10,362	231 (2.2)	888 (8.6)	1,798 (17.4)	–	–
Western fast food					513.00	**<0.001**
≥8 times	110	6 (5.5)	10 (9.1)	51 (46.4)	–	–
5–7 times	386	7 (1.8)	28 (7.3)	196 (50.8)	–	–
2–4 times	2,289	60 (2.6)	236 (10.3)	660 (28.8)	–	–
≤1 times	12,655	280 (2.2)	1,166 (9.2)	2,111 (16.7)	–	–
Spicy hot pot skewers (Malatang)					393.15	**<0.001**
≥8 times	143	7 (4.9)	19 (13.3)	54 (37.8)	–	–
5–7 times	389	6 (1.5)	32 (8.2)	181 (46.5)	–	–
2–4 times	2,172	46 (2.1)	212 (9.8)	616 (28.4)	–	–
≤1 times	12,736	294 (2.3)	1,177 (9.2)	2,167 (17.0)	–	–
Conventional Chinese hotpot (Huoguo)					572.69	**<0.001**
≥8 times	104	5 (4.8)	8 (7.7)	48 (46.2)	–	–
5–7 times	322	5 (1.6)	22 (6.8)	173 (53.7)	–	–
2–4 times	1,308	19 (1.5)	107 (8.2)	469 (35.9)	–	–
≤1 times	13,706	324 (2.4)	1,303 (9.5)	2,328 (17.0)	–	–
Steamed buns and dim sum					315.10	**<0.001**
≥8 times	144	4 (2.8)	15 (10.4)	53 (36.8)	–	–
5–7 times	569	9 (1.6)	52 (9.1)	210 (36.9)	–	–
2–4 times	1,931	33 (1.7)	198 (10.3)	563 (29.2)	–	–
≤1 times	12,796	307 (2.4)	1,175 (9.2)	2,192 (17.1)	–	–
Desserts					310.85	**< 0.001**
≥8 times	242	13 (5.4)	21 (8.7)	83 (34.3)	–	–
5–7 times	703	19 (2.7)	72 (10.2)	267 (38.0)	–	–
2–4 times	3,326	76 (2.3)	356 (10.7)	774 (23.3)	–	–
≤1 times	11,169	245 (2.2)	991 (8.9)	1,894 (17.0)	–	–

The values inside parentheses () are percent. Bold *P* values indicate statistical significance (*P* < 0.05).

**TABLE 4:** The multivariates logistic regression analysis of the association between milk tea and takeaway food behaviors and psychological symptoms status.

Groups	Only depression		Only anxiety		Comorbidity	
	OR (95% CI)	*P*-value	OR (95% CI)	*P*-value	OR (95% CI)	*P*-value
**Milk tea beverage**						
Yes	0.97 (0.77∼1.21)	0.772	1.33 (1.18∼1.50)	**<0.001**	1.16 (1.06∼1.26)	**<0.001**
No	1	–	1	–	1	–
**Takeaway food**						
Yes	1.06 (0.84∼1.34)	0.640	1.23 (1.09∼1.40)	**0.001**	1.09 (1.00∼1.20)	0.051
No	1	–	1	–	1	–

The model was adjusted for demographic characteristics and lifestyle behavior factors. Bold *P* values indicate statistical significance (*P* < 0.05).

**FIGURE 3:**
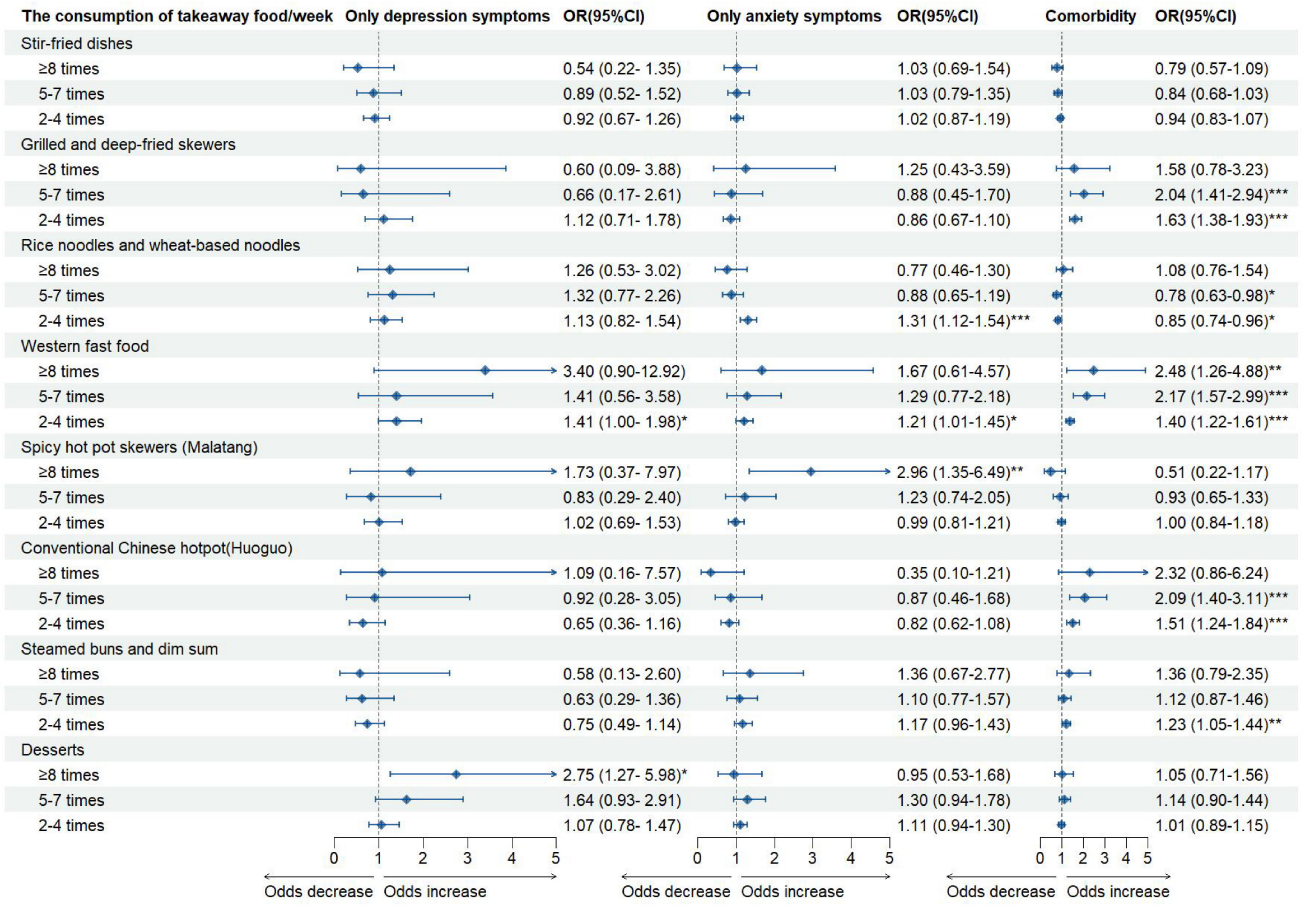
The association between takeaway food consumption and psychological symptoms status. *P < 0.05; **P < 0.01; ***P < 0.001.

For takeaway food, most consumption types were positively associated with psychological symptoms, especially comorbidity. Grilled and deep-fried skewers, western fast food, conventional Chinese hotpot (Huoguo), and steamed buns/dim sum were associated with significantly increased odds of comorbidity. Interestingly, rice noodles/wheat-based noodles consumption was positively associated with only anxiety symptoms at 2–4 times/week (OR = 1.31, 95% CI: 1.12–1.54) but inversely associated with comorbidity at both 2–4 times/week (OR = 0.85, 95% CI: 0.74–0.96) and 5–7 times/week (OR = 0.78, 95% CI: 0.63–0.98) (Figure 3).

#### Combined association of milk tea and takeaway food consumption

Interaction analysis in multivariate logistic regression models showed that the combination of milk tea and takeaway food consumption was positively associated with only anxiety symptoms (OR = 1.67, 95% CI: 1.43–1.95) and comorbidity (OR = 1.24, 95% CI: 1.11–1.38; both *p* < 0.05) (Table 5).

**TABLE 5:** The combined association of milk tea and takeaway food behaviors on psychological symptoms status.

Milk tea beverage consumption	Takeaway food consumption	Only depression		Only anxiety		Comorbidity	
		OR (95% CI)	*P*-value	OR (95% CI)	*P*-value	OR (95% CI)	*P*-value
Drinking milk tea × eating takeaway food		1.01 (0.76–1.33)	0.969	1.67 (1.43–1.95)	**<0.001**	1.24 (1.11–1.38)	**<0.001**
Drinking milk tea × non-eating takeaway food		0.86 (0.58–1.27)	0.448	1.38 (1.13–1.69)	**0.002**	1.00 (0.86–1.16)	0.965
Non-drinking milk tea × eating takeaway food		0.98 (0.72–1.34)	0.889	1.27 (1.06–1.52)	**0.008**	0.98 (0.87–1.11)	0.786
Non-drinking milk tea × non-eating takeaway food		1	–	1	–	1	–

The model was adjusted for demographic characteristics and lifestyle behavior factors. Bold *P* values indicate statistical significance (*P* < 0.05).

**FIGURE 4:**
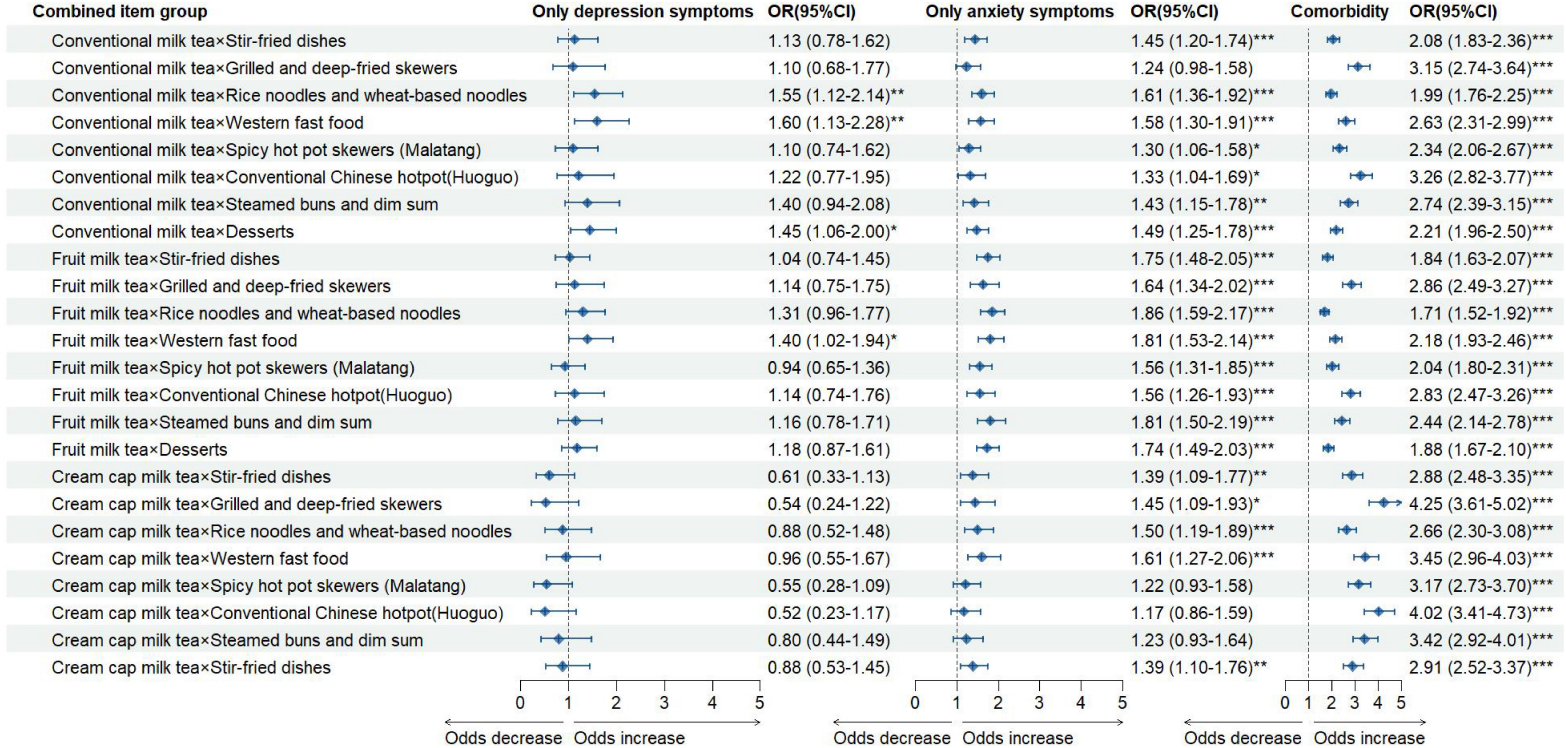
The association of various milk tea and takeaway food behavior combinations with psychological symptoms status. *P < 0.05; **P < 0.01; ***P < 0.001.

Further analyses of specific combinations revealed that, compared with students who consumed neither milk tea nor takeaway food, most combinations were significantly associated with only anxiety symptoms and comorbidity. In particular, students who preferred cream cap milk tea in combination with certain takeaway foods had the highest odds of comorbidity—especially grilled and deep-fried skewers (OR = 4.25, 95% CI: 3.61–5.02), western fast food (OR = 3.45, 95% CI: 2.96–4.03), and conventional Chinese hotpot (Huoguo) (OR = 4.02, 95% CI: 3.41–4.73) (Figure 4).

## Discussion

In this large, population-based cross-sectional study, milk tea consumption and takeaway food consumption were both associated with a higher likelihood of psychological symptoms and their comorbidity among Chinese university students. Furthermore, significant combined association of these dietary behaviors on different psychological symptom profiles were observed. These findings highlight the need for greater attention to the potential mental health risks associated with such consumption patterns.

Several previous studies have reported associations between milk tea consumption or takeaway food consumption and psychological symptoms. For example, Li et al. investigated 9,000 college students in East China and found that, compared with the reference group, consuming milk tea 4–5 times and ≥6 times per week was associated with 1.98- and 2.46-fold higher odds of comorbid overweight/obesity and depression symptoms, respectively (35). Similarly, Xu et al. reported that milk tea consumption was positively associated with depression among 31,856 Chinese students (36). However, some controversy exists. In contrast, a longitudinal observational study from Chongqing, China, found no significant multivariate association between milk tea consumption and mental health problems among 686 male university students (37). In addition, several studies have explored the relationship between takeaway food and mental health outcomes. For instance, Tan et al. reported that frequent takeaway food consumption was significantly associated with higher depression symptom scores among 6,417 Chinese freshman students (38). Likewise, Whatnall et al. found a positive dose–response relationship between takeaway food consumption frequency and psychological distress in 2,710 Australian university students (39). Notably, although some prior studies have examined the association of milk tea and takeaway food consumption on mental health in university populations, the combined association of these behaviors on psychological symptoms remains unclear. To date, only one study has reported their combined association on overweight and obesity among Chinese university students (40). Our study extends this literature by focusing on the combined association of milk tea and takeaway food consumption on psychological symptom status, particularly across different combinations of food types.

In the present study, we observed that students who engaged in both milk tea consumption and takeaway food consumption reported a higher prevalence of comorbid depression and anxiety symptoms in the univariate association analysis. Based on this, we further conducted multivariate logistic regression analyses. The results showed that, compared with individuals who did not consume milk tea or takeaway food, those who consumed milk tea had 1.33- and 1.16-fold higher odds of experiencing only anxiety symptoms and comorbidity, respectively, while those who consumed takeaway food had 1.23-fold higher odds of experiencing only anxiety symptoms. Furthermore, higher levels of different types of milk tea and takeaway food consumption were positively associated with psychological symptoms in most groups, particularly for comorbidity (see Table 5). These findings are consistent with those of Li et al. (40), Tan et al. (38), and they also confirm H1 in our study. The biological mechanisms linking milk tea and takeaway food consumption to psychological symptoms are well-documented. A growing body of research indicates that milk tea contains high levels of sugar, fat, and excessive additives (41). Similarly, most Chinese takeaway food has been reported to involve overcooking and to contain excessive oil and sodium (42, 43). Previous studies have shown that excessive intake of sugar, sodium, and oil can lead to neuroinflammation, oxidative stress, and dysregulation of neurotransmitter function in the brain, all of which are associated with mental health disorders (44–46). However, some contrasting results emerged. For instance, consuming 1–4 bottles of cream cap milk tea or ≥10 bottles of conventional milk tea was negatively associated with psychological symptoms. A plausible explanation for these unexpected findings could be reverse causation: individuals with fewer psychological symptoms may be more socially active and proactive in daily life, participating more frequently in social gatherings that lead to higher milk tea consumption (47). By contrast, those experiencing psychological issues and sleep problems may deliberately limit or avoid caffeinated, sugar-sweetened beverages, so lower intake reflects symptom-driven behavior change rather than any protective effect of milk tea itself (48). Moreover, people with a generally positive emotional state may simply derive greater enjoyment from drinking milk tea. Notably, Similarly, specific takeaway food items—such as rice noodles and wheat-based noodles—were negatively associated with comorbidity, although the reasons for this are unclear. Nevertheless, it is important to note that not all takeaway food consumption was adversely associated with psychological symptoms. Some studies suggest that healthier takeaway options—such as fruits, vegetables, rice, wheat, and soybean products—may benefit mental health by providing vitamins, dietary fiber, and high-quality fats, all of which can support the regulation of negative emotions (49, 50). The present results suggest that, future work should apply causal-inference methods and longitudinal or quasi-experimental designs to better estimate the effects of dietary intake on psychological symptoms.

Given the significant associations observed for both milk tea consumption and takeaway food consumption with psychological symptoms, we further examined their combined association on different mental health outcomes. The results showed that the significantly positive combined association was observed between these two behaviors and psychological symptoms. Compared with reference group, those who consumed both in milk tea and takeaway food have a 1.67-folds and 1.24-folds likelihood of suffering from only anxiety symptoms and comorbidity. This results verified H_2_ in the present study, see Table 5. Based on that, we furtherly explored the association between different type of milk tea and takeaway food combination. After adjusting for confounding, the results indicated that most combinations between milk tea and takeaway food have an adverse association with only anxiety symptoms and comorbidity. The H_3_ had been verified by this results. Interestingly, the combinations item including cream cap milktea, grilled and deep-fried skewers, spicy hot pot skewers (Malatang), western fast food, and Chinese hotpot (Huoguo) showed higher likelihood of suffering from comorbidity. For instance, the cream cap milk tea combined with grilled and deep-fried skewers were showed the highest odds of suffering from comorbidity (OR = 4.25, 95% CI = 3.61–5.02). Notably, no matter cream cap milk tea or those aforementioned takeaway food, they were containing more unhealthy ingredients (i.e., excessive fat, sugar, and sodium etc.) compared to their counterparts, especially for those takeaway food cooked by stir-fry or deep fried. Therefore, the present results suggest that it’s important to pay special attention to the combined consumption of high fat and high sugar milk tea and deep fried or stir-fry takeaway food.

The present study examined the association of milk tea consumption and takeaway food consumption on psychological symptoms among Chinese university students. Compared with previous research, this large-scale population-based study additionally identified an adverse combined association between these two behaviors. In contemporary, milk tea and takeaway food are highly popular among Chinese young adults. National reports indicate that nearly 81.1% of university students consume beverages, including milk tea, one to three times per week (51), and more than 70% order takeaway food on a weekly basis (52). Moreover, in 2025, the three leading food delivery platforms in China launched large-scale takeaway subsidy campaigns to consolidate their dominant market positions, further stimulating the consumption of both takeaway food and milk tea among university students. Given the high prevalence of these dietary behaviors and their potential to exacerbate the burden of psychological symptoms, there is an urgent need for targeted interventions.

This study has several notable strengths. To our knowledge, this is the first investigations to examine both the independent and combined association of milk tea and takeaway food consumption on psychological symptoms among Chinese university students. Besides, the large sample size and inclusion of multiple food categories allowed for robust statistical analyses. Nevertheless, several limitations should be acknowledged. Firstly, as a cross-sectional study, our findings can only suggest potential associations and offer limited capacity for causal inference. Second, the use of retrospective dietary epidemiology questionnaires is inevitably subject to information bias. Thirdly, we did not measure other sugar-sweetened beverages, so the specific effect of milk tea cannot be disentangled from overall beverage patterns. The inverse associations in some milk tea categories may reflect substitution or displacement (e.g., choosing milk tea over sodas/energy drinks) or co-consumption/selection effects, rather than a protective effect. Finally, some factors previously identified as being associated with psychological health, including smoking, alcohol consumption, and negative life events, were not comprehensively collected in our study. Future research should implement longitudinal cohort studies to better clarify whether milk tea and takeaway food consumption lead to psychological symptoms. It should also apply more accurate dietary assessment methods and include additional relevant factors such as smoking, alcohol consumption, other sugar-sweetened beverages consumption, and negative life events.

In conclusion, this study found that milk tea and takeaway food consumption had a significant combined association on psychological symptoms among Chinese university students. Schools should strengthen dietary and mental health education to reduce such consumption behaviors and help improve students’ psychological well-being.

## Data Availability

The (de-identified) raw data supporting the conclusions of this article will be made available by the authors upon reasonable request, in accordance with applicable ethical and privacy regulations.
